# Supplementary material for: Growth and Behavior of North American Microbes on Phragmites australis Leaves
Source: Microorganisms. 2020 May 8;8(5):690. doi: 10.3390/microorganisms8050690 (PMC7284954; doi:10.3390/microorganisms8050690)
Supplement: Supplementary file 1 [file microorganisms-08-00690-s001.zip › Supplements/Appendix S1.pdf]

Appendix S1: Supplemental Methods

Table S1: PCR Conditions and Primer Sequences

Gene Region	Primer	Primer Sequence	PCR Mastermix	PCR Conditions
SSU	NS1	5'- GTAGTCATATGCTTGTCTC -3'	10.0 uL 5x Buffer* 4.0 uL 25uM MgCl 1.0 uL 10uM dNTPs 1.0 uL 10uM NS1 1.0 uL 10uM NS4 0.25 uL Taq* 1 uL Template DNA 31.75 uL H2O	Initial denaturation: 94℃ for 2 min 40 cycles denaturation: 94℃ for 30 s annealing: 47℃ for 60 s extension: 72℃ for 80 s final extension: 72℃ for 2 min
	NS4	5'- CTTCCGTCAATTCCTTTAA -3'		
ITS	ITS1F	5'- CTTGGTCATTTAGAGGAAGTAA -3'	10.0 uL 5x Buffer* 4.0 uL 25uM MgCl 1.0 uL 10uM dNTPs 1.0 uL 10uM ITS1-F 1.0 uL 10uM ITS4 0.25 uL Taq* 1 uL Template DNA 31.75 uL H2O	Initial denaturation: 94℃ for 2 min 40 cycles denaturation: 94℃ for 30 s annealing: 51℃ for 30 s extension: 72℃ for 45 s final extension: 72℃ for 2 min
	ITS4	5'- TCCTCCGCTTATTGATATGC -3'		
LSU	LR16	5'- TTCCACCCAAACACTCG -3'	10.0 uL 5x Buffer* 4.0 uL 25uM MgCl 1.0 uL 10uM dNTPs 1.0 uL 10uM LR16 1.0 uL 10uM LROR 0.25 uL Taq* 1 uL Template DNA 31.75 uL H2O	Initial denaturation: 94℃ for 2 min 40 cycles denaturation: 94℃ for 30 s annealing: 52℃ for 30 s extension: 72℃ for 45 s final extension: 72℃ for 2 min
	LROR	5'- ACCCGCTGAACTTAAGC -3'		
16S	799F2	5'- AACMGGATTAGATACCCGG -3'	10.0 uL 5x Buffer* 4.0 uL 25uM MgCl 1.0 uL 10uM dNTPs 1.0 uL 10uM 799F2 1.0 uL 10uM 1498r 0.25 uL Taq* 1 uL Template DNA 31.75 uL H2O	Initial denaturation: 94℃ for 2 min 40 cycles denaturation: 94℃ for 30 s annealing: 49℃ for 30 s extension: 72℃ for 5 min final extension: 10 min
	1498r	5'- GGTTACCTTGTTACGACTT -3'		
CPN60[1]	H279	5'- CGCCAGGGTTTTCCCAGTCACGACGAIIIIGCIGGIGAYGGIACIACIAC -3'	10.0 uL 5x Buffer* 4.0 uL 25uM MgCl 1.0 uL 10uM dNTPs 1.0 uL 10uM H279 1.0 uL 10uM H280 0.25 uL Taq* 1 uL Template DNA 31.75 uL H2O	Initial denaturation: 94℃ for 2 min, 40 cycles denaturation: 94℃ for 60 s annealing: 52℃ for 2 min extension: 72℃ for 5 min final extension: 10 min
	H280	5'- AGCGGATAACAATTTCACACAGGAYKIYKITCICCRAAICCIGGIGCYTT -3'		

* GoTaq DNA Polymerase Kit (Promega)

[1] For sequencing, use M13F-47 = CGCCAGGGTTTTCCCAGTCACGAC and M13R-48 = AGCGGATAACAATTTCACACAGGA

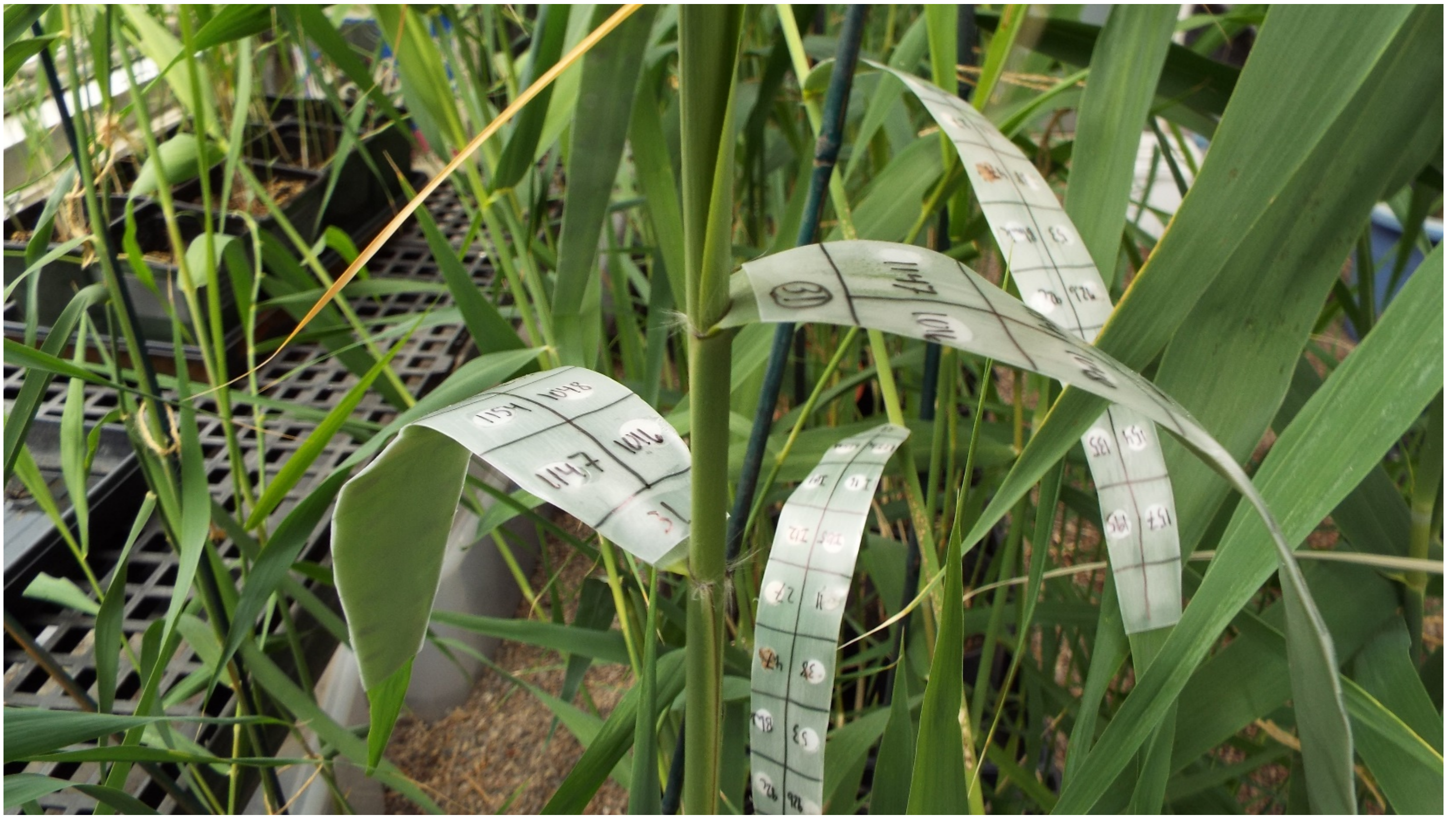

Figure S1: Mature leaf inoculation method. When saturated with live cells up to 20 disks at a time were placed inside a folded sheet of Parafilm® separated in a 2 cm grid and moistened with 5 µL of sterile molecular grade H2O. Fully loaded parafilm sheets were wrapped around the mature leaves in the upper 1/3 of 6–8-month-old plants to form a flat sleeve, with the disks on the adaxial surface, and sealed to within 1 mm of the edge of the leaf.
