# Supplementary material for: Growth and Behavior of North American Microbes on Phragmites australis Leaves
Source: Microorganisms. 2020 May 8;8(5):690. doi: 10.3390/microorganisms8050690 (PMC7284954; doi:10.3390/microorganisms8050690)
Supplement: Supplementary file 1 [file microorganisms-08-00690-s001.zip › Supplements/Appendix S2.pdf]

Appendix S2: List of microbes included in assays of mature leaves and seedlings. See full data release for raw data (https://doi.org/10.5066/P9QYEMGJ)

OTU	Kingdom	Taxon name	UNITE species hypothesis	State collected from	subsp. isolated from	Leaf types isolated from	Genbank Accession Numbers
LSU0011	Fungi	Penicillium citrinum	SH1529986.08FU	North Carolina	americanus	Healthy	MT000374,478
LSU0027	Fungi	Sarocladium kiliense	SH1706027.08FU	North Carolina	americanus	Diseased	MT000375,479
LSU0038	Fungi	Pleosporales sp.	SH1525096.08FU	North Carolina	americanus	Diseased	MT000321,376,480
LSU0047	Fungi	Chaetomium globosum	SH1615599.08FU	North Carolina	americanus, australis	Both	MT000377
LSU0053	Fungi	Paraphaeosphaeria michotii	SH1888878.08FU	North Carolina	americanus	Diseased	MT000378,481
LSU0062	Fungi	Diaporthe sp.	SH1540603.08FU	North Carolina	americanus	Diseased	MT000322,379
LSU0064	Fungi	Stagonospora sp.	SH1576715.08FU	North Carolina	americanus, australis, Gulf, Delta	Both	MT000380,482
LSU0083	Fungi	Phaeosphaeriaceae sp.	SH1525099.08FU	North Carolina	australis	Diseased	MT000381,483
LSU0093	Fungi	Arthrinium sacchari	SH1907630.08FU	Virginia	australis	Healthy	MT000329,394,490
LSU0107	Fungi	Stagonospora neglecta	SH1525143.08FU	Virginia	australis	Diseased	MT000395
LSU0119	Fungi	Cladosporium sp.	SH1572792.08FU	Virginia	americanus	Diseased	MT000323,382,484
LSU0128	Fungi	Clathrococcum neglectum	SH1547057.08FU	Virginia	americanus, australis	Diseased	MT000383
LSU0135	Fungi	Phomatospora sp.	SH1193352.08FU	Maryland	americanus, australis, Delta	Both	MT000324,384,485
LSU0151	Fungi	Pseudoanthostomella sepelibilis	SH1551327.08FU	Maryland	australis	Healthy	MT000385
LSU0154	Fungi	Stagonospora neglecta	SH1525143.08FU	Maryland	australis	Diseased	MT000386
LSU0157	Fungi	Periconia homothallica	SH1573195.08FU	Maryland	australis	Diseased	MT000325,387,486
LSU0172	Fungi	Stagonospora neglecta	SH1525143.08FU	Maryland	australis	Diseased	MT000388
LSU0182	Fungi	Sordariomycetes sp.	Unresolved at 97% threshold	New Jersey	australis, Gulf	Healthy	MT000330
LSU0195	Fungi	Trametes versicolor	SH1565939.08FU	New Jersey	australis	Diseased	MT000331,396,491
LSU0196	Fungi	Arthrinium sp.	SH1540046.08FU	New Jersey	americanus, australis, Gulf	Both	MT000332,397,492

LSU0199	Fungi	Apiosporaceae sp.	SH1540045.08FU	New Jersey	americanus, australis, Gulf	Both	N/A
LSU0203	Fungi	Cainia graminis	SH1143210.08FU	New Jersey	australis	Diseased	MT000333,398,493
LSU0207	Fungi	Colletotrichum fioriniae	SH1543706.08FU	New Jersey	australis	Diseased	MT000399,494
LSU0213	Bacteria	Bacillus megaterium	N/A	New Jersey	australis	Diseased	N/A
LSU0217	Fungi	Utrechtiana roumeguerei	SH1187174.08FU	New Jersey	americanus, australis	Both	MT000400,495
LSU0240	Fungi	Gibberella fujikuroi	SH1610159.08FU	New Jersey	americanus	Diseased	MT000496
LSU0245	Fungi	Microdochium sp.	SH1555458.08FU	New Jersey	americanus	Diseased	MT000334,402,497
LSU0246	Fungi	Microdochium sp.	SH1555458.08FU	New Jersey	americanus	Diseased	MT000403,498
LSU0254	Fungi	Cladosporium halotolerans	SH1572792.08FU	Delaware	americanus, australis, Gulf, Delta	Both	MT000326,389,487
LSU0261	Fungi	Paraphaeosphaeria sp.	SH1525447.08FU	Delaware	americanus, australis	Diseased	MT000327,390
LSU0270	Fungi	Trametopsis cervina	SH1543622.08FU	Delaware	australis	Diseased	MT000391
LSU0279	Bacteria	Bacillus megaterium	N/A	Florida	Gulf variety	Both	MN992014
LSU0281	Fungi	Purpureocillium lilacinum	SH1946757.08FU	Florida	Gulf variety, Delta	Both	MT000405,499
LSU0305	Fungi	Ascochyta manawaorae	SH1235307.08FU	Florida	Gulf variety	Diseased	MT000406
LSU0311	Fungi	Stagonospora sp.	SH1235289.08FU	Florida	Gulf variety	Both	MT000407
LSU0313	Fungi	Cucurbitariaceae sp.	SH1244117.08FU	Florida	Gulf variety	Healthy	MT000335,500
LSU0321	Fungi	Aspergillus clavatus	SH1692795.08FU	Florida	Gulf variety, Delta	Diseased	MT000336,501
LSU0361	Fungi	Colletotrichum sp.	SH1543705.08FU	Florida	Gulf variety	Both	MT000409,502
LSU0375	Fungi	Epicoccum sorghinum	SH1547058.08FU	Florida	australis, Gulf	Both	MT000410,503
LSU0376	Bacteria	Enterobacter bugandensis	N/A	Florida	Gulf variety	Diseased	MN992015
LSU0384	Fungi	Nigrospora oryzae	SH1549605.08FU	Louisiana	americanus, Gulf, Delta	Both	MT000414,506
LSU0393	Fungi	Phialemoniopsis curvata	SH1549605.08FU	Louisiana	Gulf variety	Healthy	MT000340,415,507
LSU0409	Fungi	Parasarocladium gamsii	SH1291882.08FU	Louisiana	Gulf variety	Diseased	MT000416,508
LSU0421	Fungi	Dinemasporium sp.	SH1553502.08FU	Louisiana	Gulf variety	Diseased	MT000341,417,509
LSU0498	Bacteria	Pantoea ananatis	N/A	Louisiana	Delta variety	Healthy	MN992016
LSU0528	Fungi	Phaeosphaeria sp.	SH1157055.08FU	Louisiana	Delta variety	Diseased	MT000342,420,512

LSU0558	Bacteria	Streptomycetaceae sp.	N/A	Louisiana	australis, Gulf, Delta	Diseased	N/A
LSU0560	Fungi	Cainia sp.	SH1247225.08FU	Louisiana	Gulf variety, Delta	Diseased	MT000343,421,513
LSU0565	Fungi	Microsphaeropsis sp.	SH1525446.08FU	Louisiana	Delta variety	Diseased	MT000422,514
LSU0566	Fungi	Pleospora typhicola	SH1524238.08FU	Louisiana	Delta variety	Diseased	MT000423,515
LSU0574	Bacteria	Paenibacillus polymyxa	N/A	Louisiana	americanus, Gulf, Delta	Both	MN992017
LSU0620	Fungi	Phialemoniopsis curvata	SH1541592.08FU	Louisiana	Gulf variety	Diseased	MT000418,510
LSU0629	Fungi	Fusarium sp.	SH1610165.08FU	Louisiana	Gulf variety	Diseased	MT000419,511
LSU0648	Fungi	Aspergillus ustus	SH1549902.08FU	Louisiana	Delta variety	Healthy	MT000424,516
LSU0667	Bacteria	Streptomycetaceae sp.	N/A	Louisiana	Delta variety	Diseased	N/A
LSU0677	Fungi	Microdochium sp.	SH1555458.08FU	Louisiana	Gulf variety, Delta	Both	MT000425,517
LSU0694	Fungi	Phaeosphaeria sp.	SH1151640.08FU	Connecticut	americanus	Diseased	MT000244,426,518
LSU0696	Bacteria	Rhizobium radiobacter	N/A	Connecticut	americanus	Diseased	N/A
LSU0705	Fungi	Phaeosphaeriaceae sp.	SH1525087.08FU	Connecticut	americanus	Diseased	MT000345,427,519
LSU0706	Fungi	Paraphaeosphaeria michotii	SH1888878.08FU	Connecticut	americanus, australis	Diseased	MT000428,520
LSU0711	Fungi	Stagonospora sp.	SH1576715.08FU	Connecticut	americanus, australis, Gulf	Diseased	MT000429,521
LSU0719	Fungi	Epicoccum sorghinum	SH1547058.08FU	Rhode Island	australis	Diseased	MT000430,522
LSU0722	Fungi	Stagonospora neglecta	SH1525143.08FU	Rhode Island	australis	Diseased	MT000431
LSU0731	Fungi	Acrodontium crateriforme	SH1521400.08FU	Rhode Island	australis	Diseased	MT000346,432,523
LSU0739	Bacteria	Bacillus megaterium	N/A	Rhode Island	australis	Diseased	N/A
LSU0743	Fungi	Alternaria alternata	SH2179670.08FU	Massachusetts	americanus	Healthy	MT000433
LSU0755	Fungi	Phaeosphaeria sp.	SH1525091.08FU	Massachusetts	americanus	Diseased	MT000347,434,524
LSU0760	Fungi	Nigrospora oryzae	SH1549606.08FU	Massachusetts	americanus, australis, Delta	Both	MT000435
LSU0762	Fungi	Phaeosphaeria sp.	SH1156212.08FU	Massachusetts	americanus	Diseased	MT000348,436,525
LSU0766	Fungi	Alternaria alternata	SH2179670.08FU	Massachusetts	americanus, australis, Gulf, Delta	Diseased	MT000437,437,526
LSU0773	Fungi	Biscogniauxia mediterranea	SH1554921.08FU	Massachusetts	australis	Healthy	MT000438,527
LSU0782	Fungi	Pleosporaceae sp.	SH1157091.08FU	Massachusetts	americanus, australis, Gulf, Delta	Diseased	MT000439

LSU0787	Fungi	Irpex lacteus	SH1573566.08FU	Massachusetts	australis	Diseased	MT000440
LSU0795	Fungi	Neoascochyta paspali	SH1920693.08FU	Massachusetts	americanus, australis, Gulf, Delta	Diseased	MT000441,528
LSU0801	Fungi	Stagonospora sp.	SH1576715.08FU	Maine	australis	Diseased	MT000354,452,537
LSU0807	Fungi	Didymella glomerata	SH2232036.08FU	Maine	americanus, australis	Diseased	MT000453,538
LSU0829	Fungi	Penicillium paxilli	SH1530004.08FU	Virginia	australis	Healthy	MT000404
LSU0835	Fungi	Papiliotrema pseudoalba	SH1650804.08FU	Maine	americanus	Healthy	MT000454
LSU0848	Fungi	Septoriella poae	SH1525210.08FU	Maine	americanus	Diseased	MT000455
LSU0851	Fungi	Phaeosphaeria sp.	SH1525087.08FU	Maine	americanus, australis, Gulf, Delta	Diseased	MT000355,456,539
LSU0866	Fungi	Dothioraceae sp.	Unresolved at 97% threshold	Maine	americanus	Healthy	MT000356,457,540
LSU0868	Bacteria	Streptomycetaceae sp.	N/A	Maine	americanus	Healthy	N/A
LSU0874	Fungi	Stagonospora sp.	SH1576715.08FU	Maine	americanus	Healthy	MT000357,458,541
LSU0876	Fungi	Stagonospora sp.	SH1525099.08FU	Maine	americanus	Both	MT000358,459,542
LSU0880	Fungi	Phaeosphaeriaceae sp.	SH1646332.08FU	Maine	americanus, Gulf, Delta	Diseased	MT000359,460,543
LSU0893	Fungi	Sarocladium strictum	SH1541921.08FU	Maine	americanus, australis	Both	MT000461,544
LSU0894	Fungi	Phaeosphaeriaceae sp.	SH1235297.08FU	Maine	americanus	Diseased	MT000360,462,545
LSU0913	Fungi	Daldinia childiae	SH1507871.08FU	Maine	australis	Diseased	MT000444,530
LSU0928	Fungi	Acrodontium crateriforme	SH1521400.08FU	Maine	australis	Diseased	MT000351,445,531
LSU0932	Fungi	Paraphaeosphaeria sp.	SH1525447.08FU	Maine	australis, Delta	Diseased	MT000352,446,532
LSU0936	Fungi	Phlebia subserialis	SH1544537.08FU	Maine	americanus	Healthy	MT000447
LSU0954	Fungi	Stagonospora sp.	SH1576715.08FU	Maine	americanus, australis	Diseased	MT000533
LSU0960	Fungi	Bjerkandera adusta	SH2047468.08FU	Maine	americanus	Diseased	MT000448,534
LSU0965	Fungi	Thuemenella cubispora	SH1544084.08FU	Maine	australis	Diseased	MT000449,535
LSU0976	Fungi	Phlebiopsis flavidoalba	SH1573650.08FU	Maine	australis	Both	MT000450
LSU0994	Fungi	Nigrospora oryzae	SH1549605.08FU	Maryland	australis	Diseased	MT000328,393,489
LSU1016	Fungi	Bjerkandera adusta	SH2047468.08FU	Delaware	australis	Healthy	MT000393

LSU1024	Fungi	Sordariomycetes sp.	SH1244533.08FU	Louisiana	Gulf variety	Healthy	N/A
LSU1048	Fungi	Phialemoniopsis curvata	SH1541592.08FU	Louisiana	Gulf variety	Diseased	MT000361,463,546
LSU1063	Fungi	Arthrinium sp.	SH1540046.08FU	Louisiana	australis, Gulf	Both	MT000362,464,547
LSU1108	Fungi	Dothideomycetes sp.	Unresolved at 97% threshold	Louisiana	Gulf variety	Diseased	MT000465
LSU1120	Fungi	Arthrinium sp.	SH1540046.08FU	Louisiana	Gulf variety	Diseased	MT000363,466,548
LSU1127	Fungi	Paraphaeosphaeria sp.	SH1525447.08FU	Louisiana	Gulf variety	Diseased	MT000467,549
LSU1134	Bacteria	Streptomycetaceae sp.	N/A	Louisiana	Gulf variety	Both	N/A
LSU1147	Fungi	Phaeosphaeriopsis musae	SH2100711.08FU	Louisiana	Gulf variety	Diseased	MT000364,468,550
LSU1154	Fungi	Dinemasporium sp.	SH1178094.08FU	Louisiana	Gulf variety	Diseased	MT000365,469,551
LSU1158	Bacteria	Curtobacterium sp.	N/A	Louisiana	Gulf variety	Diseased	MN992019
LSU1167	Fungi	Colletotrichum sp.	SH1543705.08FU	Louisiana	Gulf variety	Diseased	MT000470,552
LSU1175	Fungi	Pestalotiopsis sp.	SH1563658.08FU	Louisiana	americanus, australis, Gulf	Both	MT000366,471,553
LSU1179	Fungi	Cladosporium sp.	SH1572792.08FU	Louisiana	americanus, australis, Gulf	Both	MT000367,472,554
LSU1213	Fungi	Phaeosphaeriopsis musae	SH2100711.08FU	Louisiana	Gulf variety	Diseased	MT000368,473,555
LSU1218	Fungi	Bjerkandera adusta	SH2047468.08FU	Louisiana	australis, Gulf	Diseased	MT000474,556
LSU1226	Fungi	Penicillium sclerotiorum	SH1537124.08FU	Louisiana	Gulf variety	Diseased	MT000475
LSU1236	Fungi	Penicillium sclerotiorum	SH1537124.08FU	Florida	Gulf variety	Healthy	MT000338,411,504
LSU1246	Fungi	Aureobasidium pullulans	SH1674913.08FU	Florida	Gulf variety	Diseased	MT000412
LSU1247		Unknown	N/A	Florida	Gulf variety	Healthy	N/A
LSU1250	Fungi	Paraphaeosphaeria sp.	SH1525447.08FU	Florida	Gulf variety	Diseased	MT000339
LSU1284	Bacteria	Bacillus megaterium	N/A	Louisiana	Gulf variety, Delta	Both	N/A
LSU1322	Fungi	Moesziomyces bullatus	SH1509407.08FU	Massachusetts	americanus	Healthy	MT000442
LSU1329	Fungi	Niesslia sp.	SH1506678.08FU	Massachusetts	australis	Diseased	MT000350,443,529
LSU1339	Bacteria	Streptomycetaceae sp.	N/A	Maine	americanus	Both	N/A
LSU1345	Fungi	Xylaria sp.	SH1704925.08FU	Maine	australis	Diseased	MT000353,451,536

LSU1351	Fungi	Trametes versicolor	SH1565939.08FU	Louisiana	Gulf variety	Healthy	MT000476,557
LSU1358	Bacteria	Sphingomonas sp.	N/A	Louisiana	Gulf variety	Diseased	MN992020
LSU1369	Fungi	Rhinocladiella atrovirens	SH1970592.08FU	Louisiana	Gulf variety	Both	MT000477
USGS01	Bacteria	Bacillus pumilus	N/A	Ohio	australis	Diseased	N/A
USGS02	Fungi	Stagonospora neglecta	SH1525143.08FU	Ohio	australis	Diseased	MT000585,616
USGS03	Fungi	Pleosporales sp.	SH1920671.08FU	Michigan	americanus	Healthy	MT000589,619,642
USGS04	Bacteria	Bacillus pumilus	N/A	Michigan	americanus	Healthy	N/A
USGS05	Fungi	Ustilaginaceae sp.	SH1509427.08FU	Ohio	americanus	Diseased	MT000586,640
USGS07	Fungi	Gibberella fujikuroi	SH1610159.08FU	Ohio	americanus	Diseased	MT000587,617,641
USGS08	Fungi	Aureobasidium pullulans	SH1674913.08FU	Michigan	americanus	Healthy	MT000590
USGS09	Fungi	Massarinaceae sp.	SH1576715.08FU	Ohio	australis	Diseased	MT000588,618
USGS10	Bacteria	Bacillus megaterium	N/A	Michigan	australis	Diseased	N/A
USGS11	Fungi	Alternaria sp.	SH1526398.08FU	Michigan	australis	Diseased	MT000591,620
USGS12	Bacteria	Curtobacterium flaccumfaciens	N/A	Michigan	australis	Diseased	MT000612
USGS13	Fungi	Phaeosphaeriaceae sp.	SH1525087.08FU	Michigan	australis	Diseased	MT000592,621
USGS14	Fungi	Achroiostachys humicola	SH2270274.08FU	Michigan	americanus	Diseased	MT000584,615
USGS15	Fungi	Septoriella hubertusii	SH2176229.08FU	Michigan	australis	Diseased	MT000583,614,639
USGS16	Bacteria	Bacillus pumilus	N/A	Michigan	americanus	Diseased	N/A
USGS17	Fungi	Alternaria sp.	SH1526398.08FU	Michigan	americanus	Diseased	MT000593
USGS20	Fungi	Alternaria sp.	SH1526398.08FU	Indiana	americanus, australis	Diseased	MT000599,625
USGS21	Fungi	Neovossia moliniae	SH2149896.08FU	Ohio	americanus	Diseased	MT000594,643
USGS23	Fungi	Curvularia inaequalis	SH1890305.08FU	Ohio	americanus	Diseased	MT000595
USGS24	Fungi	Alternaria alternata	SH2179670.08FU	Ohio	americanus	Diseased	MT000596,622
USGS25	Fungi	Cladosporium cladosporioides	SH1572792.08FU	Ohio	americanus	Diseased	MT000597,623
USGS26	Fungi	Alternaria alternata	SH2179670.08FU	Ohio	americanus	Diseased	MT000598,624
USGS28	Fungi	Alternaria sp.	SH1526398.08FU	Michigan	australis	Diseased	MT000600,626

USGS29	Fungi	Curvularia inaequalis	SH1890305.08FU	Michigan	australis	Diseased	MT000601,627
USGS30	Fungi	Pleosporaceae sp.	SH1547057.08FU	Michigan	australis	Diseased	MT000602,628
USGS31	Fungi	Clathrococcum neglectum	SH1547057.08FU	Michigan	australis	Diseased	MT000603,629
USGS32	Fungi	Bipolaris sorokiniana	SH1526399.08FU	Michigan	australis	Diseased	MT000604,630
USGS33	Fungi	Fusarium sporotrichioides	SH2456045.08FU	Michigan	australis	Diseased	MT000605,631
USGS34	Fungi	Curvularia sp.	SH1526408.08FU	Michigan	australis	Diseased	MT000606,632
USGS35	Fungi	Alternaria alternata	SH2179670.08FU	Michigan	australis	Diseased	MT000607,633
USGS36	Fungi	Deightoniella sp.	Unresolved at 97% threshold	Michigan	australis	Diseased	MT000608,634
USGS37	Fungi	Alternaria sp.	SH1526398.08FU	Michigan	australis	Diseased	MT000635
USGS38	Fungi	Alternaria sp.	SH1526398.08FU	Michigan	australis	Diseased	MT000609,636
USGS39	Bacteria	Delftia acidovorans	N/A	Michigan	australis	Diseased	N/A
USGS40	Fungi	Stagonospora sp.	SH1576715.08FU	Michigan	australis	Diseased	MT000610,637
USGS41	Bacteria	Pectobacterium carotovorum	N/A	Michigan	australis	Diseased	MT000613
USGS42	Fungi	Fusarium sporotrichioides	SH2456045.08FU	Michigan	australis	Diseased	MT000638
