# Supplementary material for: Growth and Behavior of North American Microbes on Phragmites australis Leaves
Source: Microorganisms. 2020 May 8;8(5):690. doi: 10.3390/microorganisms8050690 (PMC7284954; doi:10.3390/microorganisms8050690)
Supplement: Supplementary file 1 [file microorganisms-08-00690-s001.zip › Supplements/Appendix S3.pdf]

Appendix S3: Literature Used in Literature Based Microbiome Comparison

Abed, R. M. M., Al-Kharusi, S., Gkorezis, P., Prigent, S., & Headley, T. (2018). Bacterial communities in the rhizosphere of *Phragmites australis* from an oil-polluted wetland. *Archives of Agronomy and Soil Science*, *64*(3), 360–370. https://doi.org/10.1080/03650340.2017.1352087

Achon, M. A., Serrano, L., Clemente-Orta, G., & Sossai, S. (2017). First Report of Maize chlorotic mottle virus on a Perennial Host, *Sorghum halepense*, and Maize in Spain. *Plant Disease*, *101*(2), 393–393.

Al-Ani, R. A., Adhab, M. A., El-Muadhidi, M. A., & Al-Fahad, M. A. (2012). Biological and Serological Identification of Barley Yellow Dwarf Virus (BYDV) and Its Distribution in Iraq. *Journal of Agricultural Science*, *4*(2). https://doi.org/10.5539/jas.v4n2p39

Al-Garni, S. (2006). Increasing NaCl - Salt Tolerance of a Halophytic Plant *Phragmites australis* by Mycorrhizal Symbiosis. *American-Eurasian Journal of Agricultural & Environmental Sciences*, *1*(2), 119–126.

Allen, W. J., DeVries, A. E., Bologna, N. A., Bickford, W. A., Kowalski, K. P., Meyerson, L. A., & Cronin, J. T. (2020). Intraspecific and biogeographic variation in foliar fungal communities and pathogen damage of native and invasive *Phragmites australis*. *Global Ecology and Biogeography*, *doi: 10.1111/geb.13097*

Angelini, P., Rubini, A., Gigante, D., Reale, L., Pagiotti, R., & Venanzoni, R. (2012). The endophytic fungal communities associated with the leaves and roots of the common reed (*Phragmites australis*) in Lake Trasimeno (Perugia, Italy) in declining and healthy stands. *Fungal Ecology*, *5*(6), 683–693. https://doi.org/10.1016/j.funeco.2012.03.001

Apinis, A. E., Chesters, C. G. C., & Taligoola, H. K. (1972a). Colonisation of *Phragmites communis* leaves by fungi. *Nova Hedwigia*, *23*, 113–124.

Apinis, A. E., Chesters, C. G. C., & Taligoola, H. K. (1972b). Microfungi Colonizing Submerged Standing Culms of *Phragmites communis* Trin. *Nova Hedwigia*, *23*, 473–480.

Apinis, A. E., Chesters, C. G. C., & Taligoola, H. K. (1975). Microfungi colonizing nodes and internodes of aerial standing culms of *Phragmites communis* Trin. *Nova Hedwigia*, *26*, 495–507.

Arthur, J. C. (1902). The Uredineæ occurring upon *Phragmites*, *Spartina*, and *Arundinaria* in America. *Botanical Gazette*, *34*(1), 1–20.

Arthur, J. C. (1912). Cultures of *Uredineae* in 1911. *Mycologia*, *4*(2), 49–65. https://www.jstor.org/stable/3753541

Baka, Z. A., & Gjærum, H. B. (1996). Egyptian Uredinales. I. Rusts on wild plants from the Nile Delta. *Mycotaxon*, *60*, 291–303.

Bán, R., Fischl, G., & Virányi, F. (1996). A spatio-temporal analysis of fungal pathogens on reed in natural habitats. *Acta Phytopathologica et Entomologica Hungarica*, *31*(3–4), 219–227.

Bán, R., Virányi, F., & Obádovics, C. (1998). Különöböző típusú nádállományok gomba okozta

beegségi. *Növénytermelés*, *47*(4), 407–420.

Bickford, W. A., Goldberg, D. E., Kowalski, K. P., & Zak, D. R. (2018). Root endophytes and invasiveness: no difference between native and non-native *Phragmites* in the Great Lakes Region. *Ecosphere*, *9*(12). https://doi.org/10.1002/ecs2.2526

Björk, A. S. (1962). Kromosomgeografi och kromosomekologi beträffande *Phragmites communis* (Chromosome geography and ecology of *Phragmites communis*). *Skrifter Utgivna Av Södra Sveriges Fiskeriförenig*, 71–81.

Borsodi, A. K., Rusznyák, A., Molnár, P., Vladár, P., Reskóné, M. N., Tóth, E. M., Sipos, R., Gedeon, G., & Márialigeti, K. (2007). Metabolic activity and phylogenetic diversity of reed (*Phragmites australis*) periphyton bacterial communities in a Hungarian shallow soda lake. *Microbial Ecology*, *53*(4), 612–620. https://doi.org/10.1007/s00248-006-9133-x

Cerri, M., Reale, L., Moretti, C., Buonaurio, R., Coppi, A., Ferri, V., Foggi, B., Gigante, D., Lastrucci, L., Quaglia, M., Venanzoni, R., & Ferranti, F. (2018). Claviceps arundinis identification and its role in the die-back syndrome of *Phragmites australis* populations in central Italy. *Plant Biosystems*, *152*(4), 818–824. https://doi.org/10.1080/11263504.2017.1347111

Chaturvedi, S., Chandra, R., & Rai, V. (2006). Isolation and characterization of *Phragmites australis* (L.) rhizosphere bacteria from contaminated site for bioremediation of colored distillery effluent. *Ecological Engineering*, *27*(3), 202–207. https://doi.org/10.1016/j.ecoleng.2006.02.008

Çikikçi, G. (2009). Yüksek Lısans tezı Tekırdağ ılı’nde tahil üretım alanlarindakı yabanci otlarda görülen vırüslerın saptanmasi üzerıne araştirmalar (Identification of viruses on weeds around the cereal fields in Tekirdağ province). *Masters Thesis*, 1–31. ???

Clay, K., Shearin, Z. R. C., Bourke, K. A., Bickford, W. A., & Kowalski, K. P. (2016). Diversity of fungal endophytes in non-native *Phragmites australis* in the Great Lakes. *Biological Invasions*, *18*(9). https://doi.org/10.1007/s10530-016-1137-y

Constantinescu, O. (1983). Deightoniella on *Phragmites*. *Proceedings of the Koninklijke Nederlandse Akademie van Wetenschappen. Series C, Biological and Medical Sciences.*, *86*, 137–141.

Corner, E. J. H. (1935). The fungi of Wicken Fen, Cambridgeshire. *Transactions of the British Mycological Society*, *19*(4), 280–287.

Crocker, E. V., Karp, M. A., & Nelson, E. B. (2015). Virulence of oomycete pathogens from *Phragmites australis*-invaded and noninvaded soils to seedlings of wetland plant species. *Ecology and Evolution*, *5*(11), 2127–2139. https://doi.org/10.1002/ece3.1468

Crous, P. W., & Groenewald, J. Z. (2013). A phylogenetic re-evaluation of Arthrinium. *IMA Fungus*, *4*(1), 133–154. https://doi.org/10.5598/imafungus.2013.04.01.13

Cunnell, G. J. (1958). On *Robillarda phragmitis* sp. nov. *Transactions of the British Mycological Society*, *4158*(4), 405–412.

Durska, B. (1969). Rozmieszeczeni w Polce kilku gatunków grzybów pasożytniczych trzciny.

(Distribution of some parasitic fungi on *Phragmites communis* Trin. In Poland). *Acta Mycologica*, *5*, 117–133.

Durska, B. (1970). Changes in the reed (*Phragmites communis* Trin.) condition caused by disease of fungal and animal origin. *Polskie Archiwum Hydrobiologii*, *17*(30), 373–396.

Ellis, M. B., Ellis, E. A., & Ellis, J. P. (1951). British marsh and fen fungi. *Transactions of the British Mycological Society*, *34*, 147–169.

Fischer, M. S., & Rodriguez, R. J. (2013). Fungal endophytes of invasive *Phragamites australis* populations vary in species composition and fungicide susceptibility. *Symbiosis*, *61*(2), 55–62. https://doi.org/10.1007/s13199-013-0261-z

Fischl, G. (1995). A balaton mocsári növénycin elóforduló mikroszkopikus gombafajok (Microscopic fungi occurring on aquatic plants of Balaton Lake). *Növényvédelem*, *31*, 229–331.

Fischl, G., Büergés, G., & Szeglet, P. (1998). Plant hygiene conditions of the reed in lake Balaton with special view of fungal diseases. *Proceedings of the 50th International Symposium on Crop Protection*, *53*(9), 1689–1699. https://doi.org/10.1017/CBO9781107415324.004

Fraser, W. P. (1919). Cultures of Heteroecious Rusts in 1918. *Mycologia*, *11*(3), 129. https://doi.org/10.2307/3753418

Fukuhara, M. (2002). Three *Phaeosphaeria* species and *Paraphaeosphaeria michotii* isolated from *Phragmites* leaves in Osaka, Japan. *Mycoscience*, *43*(5), 375–382. https://doi.org/10.1007/s102670200055

Guy, P. L., Johnstone, G. R., & Morris, D. I. (1987). Barley yellow dwarf viruses in, and aphids on, grasses (including cereals) in Tasmania. *Australian Journal of Agricultural Research*, *38*(1), 139–152. https://doi.org/10.1071/AR9870139

Harada, Y. (1987). Aecial hosts for three graminicolous *Puccinia* species (Uredinales) in Japan, with a designation of biologic forms in *Puccinia phragmitis*. *Transactions of the Mycological Society of Japan*, *28*(2), 197–208.

Harada, Y., & Hasegawa, H. (1975). Aecial states of two *Phragmites* rust fungi, *Puccinia moriokaensis* S. Ito and *P. okatamaensis* S. Ito. *Transactions of the Mycological Society of Japan*, *16*, 42–50. https://doi.org/10.1017/CBO9781107415324.004

Hipol, R. M., & Cuevas, V. C. (2014). Copper Tolerance and Copper Accumulation of Culturable Endophytic Yeasts of *Phragmites Australis* Cav . (Trin) ex Steud . From the Mine Tailings Pond in Mankayan , Benguet , Philippines. *Asian Journal of Applied Sciences*, *02*(05), 636–643.

Hodson, E. R. (1900). A New Species of *Neovossia*. *Botanical Gazette*, *30*(4), 273–274.

Ilbaği, H. (2006). Common reed (*Phragmites communis*) is a natural host of important cereal viruses in the Trakya region of Turkey. *Phytoparasitica*, *34*(5), 441–448. https://doi.org/10.1007/BF02981198

Ingold, C. T. (1954). Aquatic Ascomycetes: Discomycetes from lakes. *Transactions of the British Mycological Society*, *37*, 1–18.

Ingold, C. T. (1955). Aquatic Ascomycetes: further species from the English Lake District. *Transactions of the British Mycological Society*, *38*, 157–168.

Itō, S. (1909). On the Uredineae Parasitic on the Japanese Gramineae. *Journal of the College of Agriculture*, *3*, 180–262.

Ivanović, D, Osler, R., Katis, N., Ivanović, M., Lgnjatović, D., Ivanović, D., Osler, R., Katis, N., Ivanović, M., & Principal, D. L. (1995). *Principal maize viruses in Mediterranean countries To cite this version : HAL Id : hal-00885702 maize viruses in Mediterranean countries*. *15*, 443–446.

Ivanović, Dragica. (1992). Distribution and frequency of maize dwarf mosaic virus in Yugoslavia. *Zaštita Bilja*, *43*(1), br.199: 35-46. https://doi.org/10.1017/CBO9781107415324.004

Jiang, N., Li, J., & Tian, C. M. (2018). *Arthrinium* species associated with bamboo and reed plants in China. *Fungal Systematics and Evolution*, *2*(December), 1–9. https://doi.org/10.3114/fuse.2018.02.01

Jordá, C., Font, I., Lázaro, A., Juarez, M., Ortega, A., & Lacasa, A. (2000). New Natural Hosts of Tomato spotted wilt virus . *Plant Disease*, *84*(4), 489–489. https://doi.org/10.1094/pdis.2000.84.4.489d

Jorda, C., Osca, J. M., & Alfaro, A. (1987). Comparative study of the Spanish strains of BYDV. *Proc. 7 Th Congress of the Mediterranean Phytopathological Union (Granada, Spain)*, 106–107. https://www.unhcr.org/publications/manuals/4d9352319/unhcr-protection-training-manual-european-border-entry-officials-2-legal.html?query=excom 1989

Kanaujia, R. S., Kishore, R., & Singh, C. S. (1978). Three new fungal diseases from India. *Acta Botanica Indica*, *6*, 92–93.

Kurokawa, K., & Tojo, M. (2010). First record of *Pythium grandisporangium* in Japan. *Mycoscience*, *51*(4), 321–324. https://doi.org/10.1007/s10267-010-0041-z

Li, Y. H., Zhu, J. N., Liu, Q. F., Liu, Y., Liu, M., Liu, L., & Zhang, Q. (2013). Comparison of the diversity of root-associated bacteria in *Phragmites australis* and Typha angustifolia L. in artificial wetlands. *World Journal of Microbiology and Biotechnology*, *29*(8), 1499–1508. https://doi.org/10.1007/s11274-013-1316-2

Li, Y. H., Zhu, J. N., Zhai, Z. H., & Zhang, Q. (2010). Endophytic bacterial diversity in roots of *Phragmites australis* in constructed Beijing Cuihu Wetland (China). *FEMS Microbiology Letters*, *309*(1), 84–93. https://doi.org/10.1111/j.1574-6968.2010.02015.x

Llirós, M., Trias, R., Borrego, C., & Bañeras, L. (2014). Specific Archaeal Communities are Selected on the Root Surfaces of Ruppia spp. and *Phragmites australis*. *Wetlands*, *34*(2), 403–411. https://doi.org/10.1007/s13157-013-0507-9

Mathew, D. C., Ho, Y. N., Gicana, R. G., Mathew, G. M., Chien, M. C., & Huang, C. C. (2015). A rhizosphere-associated symbiont, *Photobacterium* spp. strain MELD1, and its targeted

synergistic activity for phytoprotection against mercury. *PLoS ONE*, *10*(3), 1–18. https://doi.org/10.1371/journal.pone.0121178

Matos, M. P. (2016). Molecular identification of rhizosphere bacteria from *Phragmites sp* . growing in constructed wetlands treating benzene derivative compounds *October 2016*. *October*.

Mazurkiewicz-Zapałowicz, K. (2010). Microscopic fungi of *Phragmites australis* in the littoral of two lakes in drawa national park (Nw Poland). *Polish Botanical Journal*, *55*(2), 381–389.

Micsinai, A., Borsodi, A. K., Csengeri, V., Horváth, A., Oravecz, O., Nikolausz, M., Reskóné, M. N., & Márialigeti, K. (2003). Rhizome-associated bacterial communities of healthy and declining reed stands in Lake Velencei, Hungary. *Hydrobiologia*, *506–509*(2001), 707–713. https://doi.org/10.1023/B:HYDR.0000008590.37567.fa

Nechwatal, J., & Mendgen, K. (2009). Evidence for the occurrence of natural hybridization in reed-associated *Pythium* species. *Plant Pathology*, *58*(2), 261–270. https://doi.org/10.1111/j.1365-3059.2008.01955.x

Nechwatal, Jan, & Lebecka, R. (2014). Genetic and phenotypic analyses of *Pythium* isolates from reed suggest the occurrence of a new species, *P. phragmiticola*, and its involvement in the generation of a natural hybrid. *Mycoscience*, *55*(2), 134–143. https://doi.org/10.1016/j.myc.2013.07.001

Nechwatal, Jan, Wielgoss, A., & Mendgen, K. (2005). *Pythium phragmitis* sp. nov., a new species close to *P. arrhenomanes* as a pathogen of common reed (*Phragmites australis*). *Mycological Research*, *109*(12), 1337–1346. https://doi.org/10.1017/S0953756205003990

Nechwatal, Jan, Wielgoss, A., & Mendgen, K. (2008). Diversity, host, and habitat specificity of oomycete communities in declining reed stands (*Phragmites australis*) of a large freshwater lake. *Mycological Research*, *112*(6), 689–696. https://doi.org/10.1016/j.mycres.2007.11.015

Nelson, E. B., & Karp, M. A. (2013). Soil pathogen communities associated with native and non-native *Phragmites australis* populations in freshwater wetlands. *Ecology and Evolution*, *3*(16), 5254–5267. https://doi.org/10.1002/ece3.900

Neubert, K., Mendgen, K., Brinkmann, H., & Wirsel, S. G. R. (2006). Only a few fungal species dominate highly diverse mycofloras associated with the common reed. *Applied and Environmental Microbiology*, *72*(2), 1118–1128. https://doi.org/10.1128/AEM.72.2.1118-1128.2006

Ondrej, M. (1984). Vyskyt imperfektnich hub rodu Deightoniella Hughes v Ceskoslovensku. *Česká Mykologie*, *38*, 39–45.

Pelaez, F., Collado, J., Arenal, F., Basilio, A., Cabello, A., Diez Matas, M. T., Garcia, J. B., Gonzalez Del Val, A., Gonzalez, V., Gorrochategui, J., Hernändez, P., Martin, I., Platas, G., & Vicente, F. (1998). Endophytic fungi from plants living on gypsum soils as a source of secondary metabolites with antimicrobial activity. *Mycological Research*, *102*(6), 755–761. https://doi.org/10.1017/S0953756297005662

Poon, M. O. K., & Hyde, K. D. (1998a). Biodiversity of intertidal estuarine fungi on *Phragmites* at Mai Po Marshes, Hong Kong. *Botanica Marina*, *41*(2), 141–155.

https://doi.org/10.1515/botm.1998.41.1-6.141

Poon, M. O. K., & Hyde, K. D. (1998b). Evidence for the Vertical Distribution of Saprophytic fungi on senescent *Phragmites australis* Culms at Mai Po Marshes, Hong Kong. *Botanica Marina*, *41*, 281–292.

Sánchez, A. (1967). The sections *Apostemum* and *Microstemum* of the genus *Vibrissea* (Fungi). *Journal of the Agricultural University of Puerto Rico*, *51*, 79–93. https://doi.org/10.1017/CBO9781107415324.004

Sauvêtre, A., & Schröder, P. (2015). Uptake of carbamazepine by rhizomes and endophytic bacteria of *Phragmites australis*. *Frontiers in Plant Science*, *6*(FEB), 1–11. https://doi.org/10.3389/fpls.2015.00083

Shearer, J. F., & Harms, N. E. (2012). *Survey for Pathogens of Phragmites in New York*. *April*, 1–10.

Shearin, Z. R. C., Filipek, M., Desai, R., Bickford, W. A., Kowalski, K. P., & Clay, K. (2018). Fungal endophytes from seeds of invasive, non-native *Phragmites australis* and their potential role in germination and seedling growth. *Plant and Soil*, *422*(1–2). https://doi.org/10.1007/s11104-017-3241-x

Soares, M. A., Li, H. Y., Kowalski, K. P., Bergen, M., Torres, M. S., & White, J. F. (2016). Functional Role of Bacteria from Invasive *Phragmites australis* in Promotion of Host Growth. *Microbial Ecology*, *72*(2), 407–417. https://doi.org/10.1007/s00248-016-0793-x

Soares, Marcos Antônio, Li, H. Y., Kowalski, K. P., Bergen, M., Torres, M. S., & White, J. F. (2016). Evaluation of the functional roles of fungal endophytes of *Phragmites australis* from high saline and low saline habitats. *Biological Invasions*, *18*(9), 2689–2702. https://doi.org/10.1007/s10530-016-1160-z

Sutton, B. C., & Alcornb, J. L. (1974). Neottiosporina. *Australian Journal of Botany*, *22*, 517–530.

Taligoola, H. K., Apinis, A. E., & Chesters, C. G. C. (1972). Microfungi colonising collased aerial parts of *Phragmites communis* Trin. in water. *Nova Hedwigia*, *23*, 465–472.

Terui, M., & Harada, Y. (1974). An ovaricolous smut of reeds from Japan, Neovossia danubialis Savulescu. *Transactions of the Mycological Society of Japan*, *15*(3), 215–217.

Tokuyama, T., Mine, A., Kamiyama, K., Yabe, R., Satoh, K., Matsumoto, H., Takahashi, R., & Itonaga, K. (2004). Nitrosomonas *communis* strain YNSRA, an ammonia-oxidizing bacterium, isolated from the reed rhizoplane in an aquaponics plant. *Journal of Bioscience and Bioengineering*, *98*(4), 309–312. https://doi.org/10.1016/s1389-1723(04)00288-9

Van Ryckegem, G., Gessner, M. O., & Verbeken, A. (2007). Fungi on leaf blades of *Phragmites australis* in a brackish tidal marsh: Diversity, succession, and leaf decomposition. *Microbial Ecology*, *53*(4), 600–611. https://doi.org/10.1007/s00248-006-9132-y

Van Ryckegem, Gunther, & Verbeken, A. (2005a). Fungal diversity and community structure on *Phragmites australis* (Poaceae) along a salinity gradient in the Scheldt estuary (Belgium). *Nova Hedwigia*, *80*(1–2), 173–197. https://doi.org/10.1127/0029-5035/2005/0080-0173

Van Ryckegem, Gunther, & Verbeken, A. (2005b). Fungal ecology and succession on *Phragmites australis* in a brackish tidal marsh. II. Stems. *Fungal Diversity*, *20*, 209–233.

Ważny, J., & Wytwer, T. (1963). Badania nad odpornością trzciny (*Phragmites communis* Trin.) na działanie grzybów niszczących drewno. *Folia Forestalia Polonica, Seria B*, *5*, 171–194.

White, J. F., Kingsley, K. I., Kowalski, K. P., Irizarry, I., Micci, A., Soares, M. A., & Bergen, M. S. (2018). Disease protection and allelopathic interactions of seed-transmitted endophytic pseudomonads of invasive reed grass (*Phragmites australis*). *Plant and Soil*, *422*, 195–208. https://doi.org/10.1007/s11104-016-3169-6

Wielgoss, A., Nechwatal, J., Bogs, C., & Mendgen, K. (2009). Host plant development, water level and water parameters shape *Phragmites australis*-associated oomycete communities and determine reed pathogen dynamics in a large lake. *FEMS Microbiology Ecology*, *69*(2), 255–265. https://doi.org/10.1111/j.1574-6941.2009.00701.x

Wirsel, S. G.R., Leibinger, W., Ernst, M., & Mendgen, K. (2001). Genetic diversity of fungi closely associated with common reed. *New Phytologist*, *149*(3), 589–598. https://doi.org/10.1046/j.1469-8137.2001.00038.x

Wirsel, Stefan G.R. (2004). Homogenous stands of a wetland grass harbour diverse consortia of arbuscular mycorrhizal fungi. *FEMS Microbiology Ecology*, *48*(2), 129–138. https://doi.org/10.1016/j.femsec.2004.01.006

Wirsel, Stefan G.R., Runge-Froböse, C., Ahrén, D. G., Kemen, E., Oliver, R. P., & Mendgen, K. W. (2002). Four or more species of Cladosporium sympatrically colonize *Phragmites australis*. *Fungal Genetics and Biology*, *35*(2), 99–113. https://doi.org/10.1006/fgbi.2001.1314

Wong, M. K. M., & Hyde, K. D. (2001). Diversity of fungi on six species of Gramineae and one species of Cyperaceae in Hong Kong. *Mycological Research*, *105*(12), 1485–1491. https://doi.org/10.1017/S0953756201004695

Wu, T., Xu, J., Xie, W., Yao, Z., Yang, H., Sun, C., & Li, X. (2018). Pseudomonas aeruginosa L10: A hydrocarbon-degrading, biosurfactant-producing, and plant-growth-promoting endophytic bacterium isolated from a Reed (*Phragmites australis*). *Frontiers in Microbiology*, *9*(MAY), 1–12. https://doi.org/10.3389/fmicb.2018.01087
